# Risk of Recurrent and Frequent Preterm Birth Among Women With Mitral Valve Prolapse: A Systematic Review and Meta-Analysis

**DOI:** 10.7759/cureus.74866

**Published:** 2024-11-30

**Authors:** Kathrina Antheia M Dimaano, Nensi Shah, Osamah AlQassab, Zainab Al-Sulaitti, Bhavana Nelakuditi, Bindu Jyothi Dandamudi, Safeera Khan

**Affiliations:** 1 Obstetrics and Gynecology, California Institute of Behavioral Neurosciences & Psychology, Fairfield, USA; 2 Internal Medicine, California Institute of Behavioral Neurosciences & Psychology, Fairfield, USA; 3 Neuropsychiatry, California Institute of Behavioral Neurosciences & Psychology, Fairfield, USA

**Keywords:** gynecology and obstetrics, mitral valve prolapse, obstetric outcomes, premature birth, preterm delivery

## Abstract

Preterm delivery remains a prominent problem in obstetrics with significant adverse implications for both mothers and the offspring. The incidence of mitral valve prolapse (MVP) in women of childbearing age has raised concerns about pregnancy and pregnancy connotations. The objective of this systematic review and meta-analysis is to help in understanding the plausibility of the association between MVP and preterm birth in women with a history of frequent deliveries. Following the Preferred Reporting Items for Systematic Reviews and Meta-analyses (PRISMA) guidelines, we searched the databases, including PubMed, Embase, Cochrane Library, Web of Science, and Scopus, for studies published in the period 1999 through 2024. Inclusion criteria consisted of studies such as cohort, cross-sectional, and case-control studies related to women diagnosed with MVP out of a total number of 1,029 articles found. Overall, a total of 19 studies were included in this review, with 3 of which were considered for further meta-analysis. It was revealed in the analysis that there was an association between MVP and preterm delivery when the severity of MVP was at Types II and III. The evidence also underscores the importance of both follow-up and preemptive measures among women suffering from MV prolapse for improved maternal and neonatal outcomes. The average Z-value (4.47) and p-value (0.00) for the test for overall effect size indicate the presence of a high correlation between MVP and premature delivery, suggesting satisfactory statistics on the association. The findings do indicate that MVP is a risk factor for preterm delivery (pooled ES = 0.24, 95% CI = 0.14 to 0.35, P <0.001).

## Introduction and background

Among the young women population, the rising cases of mitral valve prolapse (MVP) are fast becoming a public health issue, especially as far as pregnancy outcomes are concerned. Metabolic, which results from an abnormality of valvular heart disease, defined by systolic displacement of one or both anterior mitral valve leaflets into the left atrium, is known to affect a large section of the world’s population, with varying reports on demographic variations in different regions [1,2]. Even though the condition is benign in nature and resolved without intervention in the majority of cases, its consequences for cardiac events have been reviewed comprehensively [3]. Aside from this, however, there are still very few studies that have looked at how MVP affects obstetric outcomes in particular concerning preterm labor. A premature birth is defined as delivery for a gestational age of less than 37 weeks, which is ranked as one of the important causes of morbidity and mortality in newborns [4,5]. For these reasons, therefore, the need to look for the factors that could account for some of the increased premature births is very high in perinatal studies. In women’s studies, one of the areas that caught the researcher’s attention was the relation of MVP with the risk of childbirth. The purpose of this work was to gather and present data with particular regard to the complexity of the research techniques to facilitate the analysis, synthesis, and evaluation of the research. This method addressed the adequacy and validity of the findings. Thus, the point in defense of women suffering from MVP is that some studies have shown that women with either a tumor or other forms of valve heart disease do poorly during pregnancy [6,7]. It is also worth studying the association of MVP with preterm labor since this adversely influences the health and development of the babies. In addition, it has been noted that the occurrence of recurrent and multiple preterm deliveries in an individual patient warrants some attention, as these cases pose a greater risk to the health of both the mother and the child, and the risk factors for such occurrences may differ from the risk factors for single preterm events [8,9].

General objectives

This research sought to assess the studies on the link between MVP and repeated, frequent premature births by conducting a thorough review and meta-analysis. Specifically, it seeks to determine the parameters of the studies included in the meta-analysis, such as specific study designs, sample sizes, locales, and key findings. Additionally, the study aims to assess the clinicodemographic characteristics of the patients in these studies to better understand how MVP is associated with recurrent and frequent preterm births. Finally, the study will explore how MVP predicts the recurrence and frequency of preterm births among women by conducting a comprehensive review of the data reported in various studies and literature published also between 1999 and 2024.

## Review

Methods

This study (study protocol: PROSPERO CRD42024530485) implemented a systematic review and meta-analysis, adhering to the PRISMA (Preferred Reporting Items for Systematic Reviews and Meta-analyses) Guidelines 2020 [10].

Selection and data collection process

We reviewed the titles and abstracts identified during the search process as mentioned in the search sources and strategy section. Full papers were retrieved for further examination. The assessment involved reviewing works deemed potentially relevant by all the researchers involved to ensure conflicts were resolved. We individually reviewed publications meeting the criteria. Any disagreements were resolved through consensus with help from a research assistant.

Inclusion and exclusion criteria

This systematic review included analysis and interpretation of the studies mentioned below done from 1999 until 2024, including cohort studies, cross-sectional studies, and case controls. Their central objective was to explore the association of MVP with the occurrence and the recurrence of preterm births. Hence, case series, conference proceedings, and any non-English papers were excluded. Women of reproductive age suffering from MVP were the target population defined in this review regardless of place or nationality. In terms of diagnostic uniformity, the echocardiographic diagnosis of exit strategy must be conducted in accordance with internationally acceptable parameters for the American Heart Association and American College of Cardiology. The cohort comprised females with different degrees of prolapse and regurgitation and those with cardiac dysrhythmias. The outcome measure in this research is the incidence of preterm birth, defined as the spontaneous or medically indicated expulsion of a fetus before 37 weeks of pregnancy have been completed. From this perspective, the measure is actually quite important in examining the relation between MVP and preterm birth risk.

Search sources and strategy

The retrieval of relevant articles was optimized by searching key databases such as PubMed, Embase, Cochrane Library, Web of Science, and Scopus. The search employed a combination of medical subject heading (MeSH) terms and free-text keywords. In order to test the specificity and sensitivity of the strategy, the search phrases included “MVP”, “preterm delivery”, “premature birth”, “preterm birth”, “preterm labor”, “recurrent pregnancy loss”, “pregnancy complications”, “cardiovascular”, “obstetric factors”, “pregnancy outcome” as well as any derivatives. These terms were combined with each other using Boolean logic in an appropriate manner, that is, “AND” and “OR”. The search strategy entailed keywords for novel clinical features or categories relating to MVP and preterm delivery, in light of the evolution of terminology in medical literature. The reference lists of these studies and relevant reviews were also explored manually by the researchers. Their specific shares were put in a password-protected Microsoft Excel document for the purposes of analysis in order to achieve completeness in the review. An all-inclusive search for bibliographic material was conducted using references in the listed papers only. It was not possible to search and inspect studies or journals physically.

The following information was collected with the help of a standard data extraction form and an Excel sheet with password protection: study title, name of the first author, year of publication, type of study, URLs/DOIs of the study, and details. It was the responsibility of the principal author to carry out the data elicitation. It was guaranteed that any ambiguity found in the data was resolved by checking the extraction form of every item against the others. The intervention of an unbiased third party helped in the resolution of conflicts. The received data were processed and analyzed using SPSS (Statistic Package for Social Science) version 29. All of the strategies of search, used databases, and numbers of papers identified per database are shown in Table 1.

**Table 1:** Number of papers identified and keywords used for strategies of search

Database Used	Keywords	Number of Studies
PubMed	Mitral Valve Prolapse AND Preterm Birth	153 studies
	Mitral Valve Prolapse AND Recurrence	107 studies
	Preterm Birth AND Pregnancy Complications, Cardiovascular	61 studies
	Mitral Valve Prolapse AND Pregnancy Outcome	30 studies
Embase	Mitral Valve Prolapse AND Preterm Birth	141 studies
	Mitral Valve Prolapse AND Recurrent Pregnancy Loss	83 studies
	Preterm Labor AND Pregnancy Complication	32 studies
	Mitral Valve Prolapse AND Pregnancy Outcome	23 studies
Cochrane Library	Mitral Valve Prolapse AND Preterm Birth	19 studies
	Mitral Valve Prolapse AND Pregnancy Outcome	15 studies
	Preterm Birth AND Pregnancy Complications	11 studies
Web of Science	Mitral Valve Prolapse AND Preterm Birth	79 studies
	Mitral Valve Prolapse AND Recurrent Birth	53 studies
	Preterm Birth AND Pregnancy Complications	32 studies
	Mitral Valve Prolapse AND Pregnancy Outcome	19 studies
Scopus	Mitral Valve Prolapse AND Preterm Birth	81 studies
	Mitral Valve Prolapse AND Recurrent Pregnancy	45 studies
	Preterm Birth AND Pregnancy Complications	26 studies
	Mitral Valve Prolapse AND Pregnancy Outcome	19 studies

Quality appraisal

We employed the Cochrane Risk of Bias (RoB) 2.0 tool in order to assess potential bias in the studies being reviewed. Moreover, a meta-bias analysis involved a forest plot method of discernible in published findings. Forest plots are used in meta-analyses in lieu of textual summation of findings of studies addressing a particular clinical question, as they represent effect sizes of individual studies and their 95% confidence limits, presenting either odds ratios (ORs) or risk ratios or mean differences. In addition to applying funnel plot analysis and performing Egger’s regression test, we used the Newcastle-Ottawa scale to judge their bias risk in non-randomized studies. This evaluation was of clear focus on the non-randomized case-control and cohort studies that were reviewed and meta-analyzed in this systematic review rather than literature reviews.

Measure of Treatment Effect

The inverse variance random-effects meta-analysis was used in the analysis of treatment effect measures in the study. It is also done so as to pool the odd ratios of the different outcome measures that index the MVP and preterm births. Any p-value of less than 0.05 was interpreted as one that, in relation to the null hypothesis, was either in relation to the alternative or was considered to be strikingly significant.

Assessment of Heterogeneity

Furthermore, to measure the heterogeneity of the studies, we employed I^2^ and Cochran’s Q, which evaluates variation across studies. In order to prevent bias, we checked publication bias through I2 statistics and Chi-square tests. In this section, the ethnographic inquiries were carried out under the Cochrane Handbook’s guidelines. Applying SPSS version 29, we proceeded to seek a forest plot of the various components presented each of them in relation to the primary treatment being evaluated in this study. This bias analysis determined a level of statistical significance that had a value of 0.05 CI. This entailed the utilization of the findings that had been obtained, which had the most logical statements, and systematically explaining them qualitatively. Such differences in this study were assessed; in addition to informing each of the individual studies’ scope and results, the input-throughput-output model was used to study a variety of measures and definitions aimed at the overall association of the MVP with preterm birth incidence and recurrence. We included a few studies, which is why subgroup analyses were not carried out.

Results

**Figure 1:**
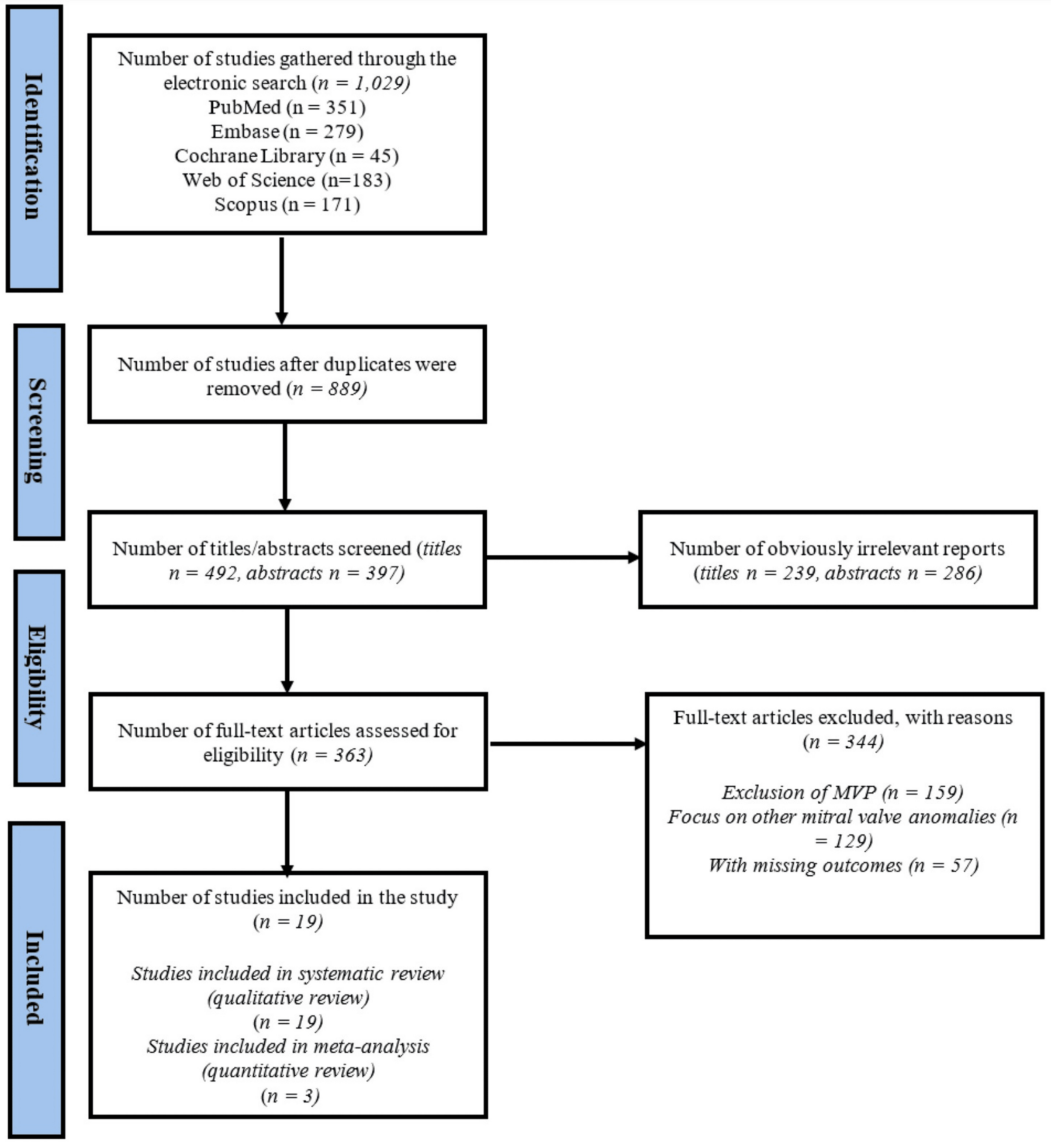
PRISMA flow diagram showing the number of studies from the electronic search up to the final inclusion after thorough screening and multiple evaluations. PRISMA: Preferred Reporting Items for Systematic Reviews and Meta-analyses; MVP: mitral valve prolapse

We initially identified 1,029 articles through our primary search. After removing 152 duplicates, 889 articles remained. These articles went through a thorough screening, where their titles and abstracts were reviewed as needed, excluding 525 articles. The abstracts of the remaining 364 articles were then reviewed, and 363 articles were chosen for full-text review. In the end, 19 articles were selected for inclusion in the study. To be specific, all 19 studies were included in the systematic review, whereas 3 of them were included in the meta-analysis. The study selection process is illustrated in the PRISMA flowchart (Figure 1).

Study characteristics

This study comprises initially of six review articles [3-8], one population-based registry study [9], one retrospective cohort study [11], two nationwide population-based cohort studies [12,13], one prospective cohort study [14], one retrospective comparative study [15], one cross-sectional observational study [16], one retrospective multi-center case series [17], one prospective registry [18], one prospective multi-center study [19], then followed by additional three review articles [20-22]. These articles consisted mainly of the significant results between the analysis of the studies regarding the relationship between MVP and preterm delivery. The participants were mainly affected by MVP with a mean age of between 18 years old and 54.7 ± 10 years. Finally, the participants’ demographics differently range based on designs of the studies. All the studies were conducted using a strong data set and method to investigate the effect of MVP in pregnancy, thus adding knowledge to its effect on maternal and neonatal health. The characteristics of the study specifically the types of the studies, results, and conclusions which are included in the systematic review are summarized in Table 1.

**Table 2:** Descriptive details of the included studies MVP: mitral valve prolapse; N/A: not available

First Author, Year	Sample Size	Mean Age	Study Design	Results	Conclusion
Delling and Vasan 2014 [3]	N/A (Review)	N/A	Review	MVP affects 2-3% of the general population, with significant risks of mitral regurgitation, heart failure, and sudden death in severe cases.	MVP is a progressive disease, often requiring long-term monitoring and early intervention to prevent severe complications.
Ohuma et al., 2023 [4]	679 data points from 103 countries	N/A	Systematic analysis	Global preterm birth rates remained steady from 2010 to 2020, with the highest burden in Southern Asia and Sub-Saharan Africa. The total number of preterm births in 2020 was 13.4 million, with 55.6% occurring in Southern Asia and Sub-Saharan Africa.	Preterm birth remains a global health issue, with little change over the last decade. The focus should be on prevention and improving data quality for better intervention outcomes.
Quinn et al., 2016 [5]	N/A	N/A	Review and guidelines	Preterm birth is a significant contributor to neonatal mortality, with 15 million infants born preterm annually. The guidelines discuss the definition, pathophysiology, and classification of preterm birth.	Accurate and standard data collection is essential to study preterm birth, particularly in vaccine trials. Clear definitions and guidelines are necessary for reliable data analysis.
Yuan and Yan, 2016 [6]	N/A (Review)	N/A	Review article	MVP is generally well tolerated during pregnancy, but serious complications such as arrhythmia and endocarditis may arise in some cases.	Non-myxomatous MVP presents a little obstetric risk, while myxomatous MVP requires more careful monitoring.
Anthony et al., 2016 [7]	Various cases (review study)	N/A	Review of valvular heart disease in pregnancy	Rheumatic mitral stenosis is poorly tolerated during pregnancy, leading to complications such as heart failure.	Rheumatic mitral stenosis poses significant risks and requires a multidisciplinary approach to management.
Behrman and Butler [8]	Studies looking at the costs associated with premature infants, large-scale research in the USA, such as state-level, insurance-based, and neonatal network studies: 1,000 premature infants. Smaller studies (specific hospitals or conducted outside the U.S.): 105 infants preterm birth outcomes: over 20,000 infants	N/A	Literature review	Preterm children with low birth weights have lower cognitive scores compared to full-term counterparts.	Preterm birth and low birth weight are associated with cognitive disadvantages in young adulthood.
Tingleff et al., 2022 [9]	213,335 women	N/A	Population-based registry study	Preterm birth rates: 5.6% for first births and 3.7% for second births. Strong association between preterm first birth and preterm second birth, with placental disorders contributing significantly to recurrent preterm birth.	A history of preterm first birth is a major risk factor for subsequent preterm births, especially with placental disorders.
Wilkie et al., 2022 [11]	23,000 (MVP)/13,597,427 (no MVP)	34 ± 6 years (MVP)/30 ± 6 years (no MVP)	Retrospective cohort study	MVP pregnancies showed a higher risk of preterm delivery (aOR 1.21, 95% CI: 1.02–1.44) and maternal mortality (HR 5.13, 95% CI: 1.09–24.16).	MVP is associated with higher risks of preterm delivery and negative obstetric outcomes.
Chen et al., 2011 [12]	3,104 (MVP)/12,245 (no MVP)	N/A	Nationwide population-based cohort study	Increased risk of preterm birth in MVP mothers (OR 1.54, p = 0.001)	MVP significantly raises the likelihood of preterm birth, highlighting the need for targeted obstetric care.
Nkomo et al., 2006 [13]	615 (valve disease)/28,412 (total subjects)	18 to 44-year-olds and 75 years and older group	Nationwide population-based cohort study	Valvular diseases are more common in older populations, with a significant increase in moderate-to-severe cases (11.7%) among those over 75.	Age significantly affects the prevalence and severity of valvular diseases like MVP.
Freed et al., 1999 [14]	84 (MVP)/3,491 (with echocardiograms)	54.7 ± 10 years	Prospective cohort study (Framingham Heart Study)	MVP subjects did not show increased risks for atrial fibrillation, heart failure, syncope, or cerebrovascular disease.	MVP is not significantly associated with major cardiac events in the Framingham cohort.
Lerman et al., 2013 [15]	390 (MVP)/233,194 (total deliveries)	N/A	Retrospective comparative study	MVP was not found to be an independent risk factor for cesarean section (OR 1.05, 95% CI: 0.79–1.37, p = 0.74).	MVP is not associated with cesarean section risks but is linked to advanced maternal age and hypertensive disorders.
Özdemir et al., 2021 [16]	39 (joint hypermobility)/42 (controls)	20.5 ± 1.1 years/20.6 ± 1.2 years	Cross-sectional observational study	No significant differences between joint hypermobility subjects and controls in MVP occurrence.	Joint hypermobility is not associated with increased MVP prevalence.
Sabbag et al., 2024 [17]	18 women with arrhythmic MVP	N/A	Retrospective multi-center case series	72% of ventricular arrhythmias occurred during pregnancy or the postpartum period.	Arrhythmic MVP increases the risk of ventricular arrhythmias, especially during pregnancy.
Van Hagen et al., 2018 [18]	390 (rheumatic mitral valve disease)/2,966 (total subjects)	28.9 ± 6 years	Prospective registry	23% of women with moderate mitral stenosis experienced heart failure during pregnancy.	Rheumatic mitral valve disease increases the risk of heart failure and poor obstetric outcomes.
Tsiaras and Poppas 2009 [19]	46 pregnancies	N/A	Prospective multi-center study	Mitral stenosis significantly impacts maternal outcomes, with pulmonary edema and arrhythmias as key complications.	Close monitoring and multidisciplinary care are necessary for pregnant women with mitral stenosis to avoid complications.
Levine et al., 2015 [20]	Review article	N/A	Review article	Mitral valve plasticity and disease progression are linked to structural and mechanobiological interactions.	Mitral valve diseases are dynamic, and early detection and intervention could help prevent severe complications.
Adamson and Nelson-Piercy 2008 [21]	N/A (Review)	N/A	Review	Arrhythmias are common in pregnancy, with supraventricular tachycardia and atrial fibrillation as the most common.	Careful monitoring and treatment of arrhythmias during pregnancy are crucial for minimizing risks.
Luthra et al., 2017 [22]	N/A (Review)	N/A	Review	Focuses on anesthesia and maternal outcomes for pregnant women with heart disease, highlighting the hemodynamic changes during labor and delivery. Properly administered anesthesia can reduce maternal and neonatal mortality.	Pregnant women with heart disease require special anesthetic management to mitigate the risks of cardiac decompensation during labor and delivery.

The risk stratification and risk perception processes may also become more manageable for women suffering from MVP, according to the findings of the study. This could prompt monitoring and intervention strategies aimed at adverse pregnancy outcomes and the two condition-related risks as well. This is particularly critical bearing in mind the effects of childbirth on the newborns, such as breathing difficulties and even the possibility of death. Nevertheless, the research addresses this gap since it investigates the factors that lead to preterm birth, an issue that has been largely overlooked among women with MVP. The comprehensiveness and applicability of study results are argued by the adoption of the PRISMA-2020 screening tool, which also sets a new research norm in the field. Thus, going through the data, it is possible to create abstractions of how MVPs may relate to pregnancy outcomes. This research is a means of moving forward in the sense that new avenues of variation can be presented regarding healthcare methods. The practical implications of this research are likely to assist healthcare policymakers and practitioners in developing interventions and policies that are likely to promote the well-being of women with MVPs. In addition, such results could influence those public health programs designed to decrease the prevalence and also the risks of associated conditions revolving around delivery. This study is a step further toward the enhancement of both the mother and infant welfare by advancing the knowledge and control of factors related to MVP risk during pregnancy. It highlights the relevance of the present research in the scope of health care and investigation.

Risk of bias in individual studies

**Figure 2:**
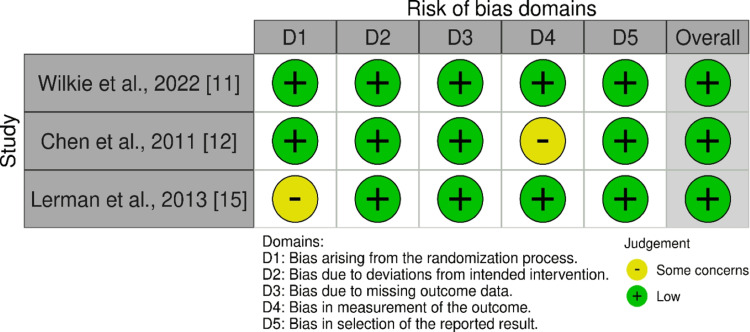
Risk-of-bias (RoB) assessment of the studies included in the meta-analysis using Cochrane Library’s RoB 2.0 tool D1: Bias arising from the process of randomization; D2: Bias due to deviations from the intervention intended; D3: Bias due to missing outcome data; D4: Bias in outcome measurement; D5: Bias in reported result selection; Judgment: (-): some concerns (color yellow); (+): low (color green).

We employed the RoB 2.0 tool available in the Cochrane Library, which helps assess the RoB in the included studies. The assessment of quality according to the Newcastle-Ottawa scale is presented in Table 3. Besides, the Cochrane RoB graph has been additionally explained in Figure 2. By and large, these studies exposed little RoB.

**Table 3:** The Newcastle-Ottawa scale used in evaluating bias among the included studies (☆) indicates a low-quality score in the respective category; (☆☆) indicates a moderate-quality score; (☆☆☆) indicates a high-quality score.

Author	Selection	Comparability	Outcomes	Total Score (maximum of 9 stars)
Delling and Vasan 2014 [3]	☆☆☆☆	☆☆	☆☆☆	☆☆☆☆☆☆☆☆☆
Ohuma et al., 2023 [4]	☆☆☆☆	☆☆	☆☆☆	☆☆☆☆☆☆☆☆☆
Quinn et al., 2016 [5]	☆☆☆☆	☆☆	☆☆	☆☆☆☆☆☆☆☆
Yuan and Yan, 2016 [6]	☆☆☆	☆	☆☆☆	☆☆☆☆☆☆☆
Anthony et al., 2016 [7]	☆☆☆☆	☆	☆☆	☆☆☆☆☆☆☆
Behrman and Butler, 2007 [8]	☆☆☆☆	☆	☆☆	☆☆☆☆☆☆☆
Tingleff et al., 2022 [9]	☆☆☆☆	☆☆	☆☆☆	☆☆☆☆☆☆☆☆☆
Wilkie et al., 2022 [11]	☆☆☆☆	☆☆	☆☆☆	☆☆☆☆☆☆☆☆☆
Chen et al., 2011 [12]	☆☆☆☆	☆☆	☆	☆☆☆☆☆☆☆
Nkomo et al., 2006 [13]	☆☆☆☆	☆☆	☆☆☆	☆☆☆☆☆☆☆☆☆
Freed et al., 1999 [14]	☆☆☆	☆	☆	☆☆☆☆☆
Lerman et al., 2013 [15]	☆☆☆	☆☆	☆☆☆	☆☆☆☆☆☆☆☆
Özdemir et al., 2021 [16]	☆☆☆	☆	☆☆	☆☆☆☆☆☆
Sabbag et al., 2024 [17]	☆☆	☆	☆	☆☆☆☆
Van Hagen et al., 2018 [18]	☆☆☆☆	☆	☆☆☆	☆☆☆☆☆☆☆☆
Tsiaras and Poppas, 2009 [19]	☆☆☆	☆☆	☆☆	☆☆☆☆☆☆☆
Levine et al., 2015 [20]	☆☆☆	☆	☆☆	☆☆☆☆☆☆
Adamson and Nelson-Piercy, 2008 [21]	☆☆☆	☆	☆☆	☆☆☆☆☆☆
Luthra et al., 2017 [22]	☆☆☆	☆	☆☆	☆☆☆☆☆☆

**Figure 3:**
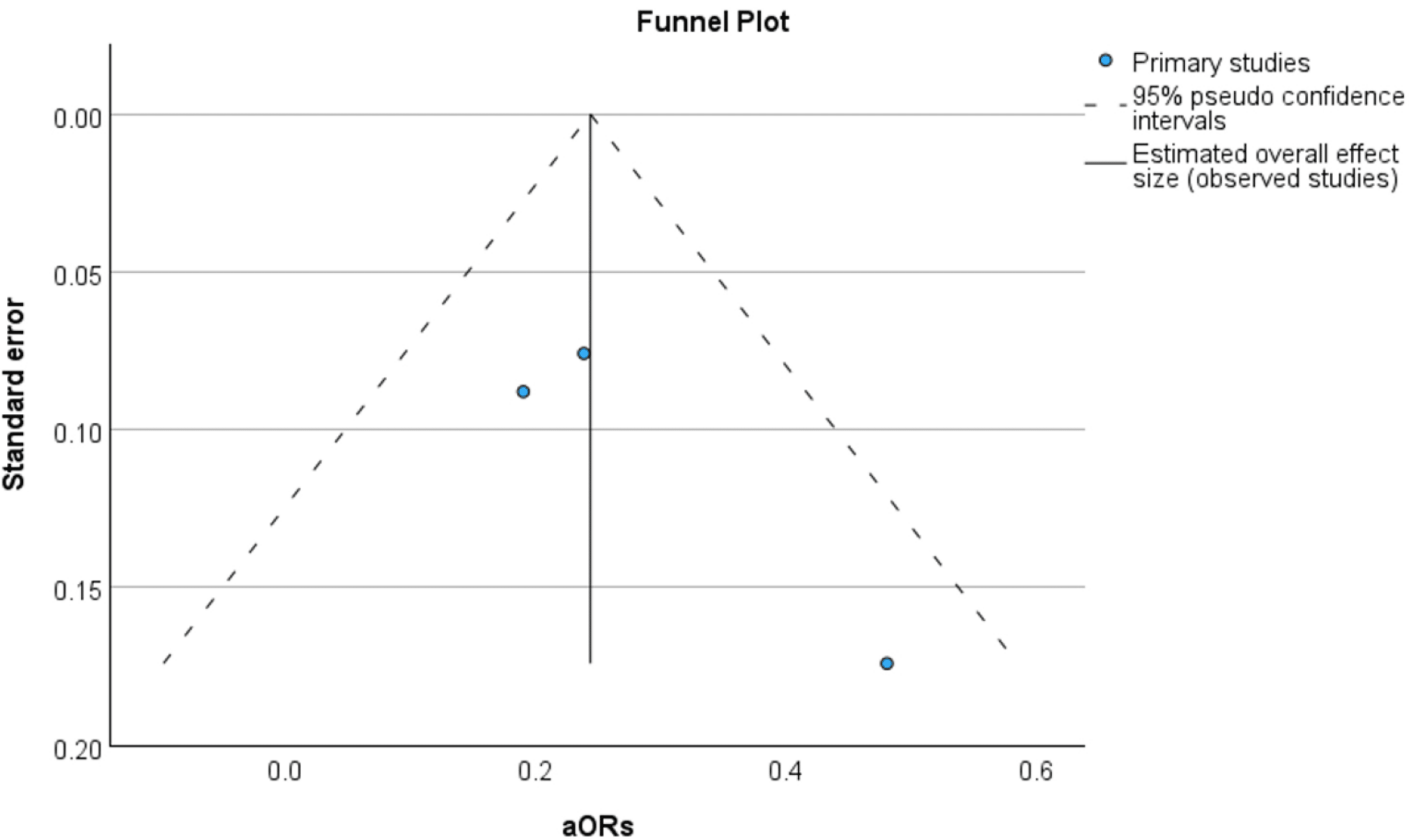
Funnel plot using the studies included in the meta-analysis Funnel plot used for the studies of Wilkie et al., 2022 [11], Chen et al., 2011 [12], and Lerman et al., 2013 [15]. aOR: adjusted odds ratio

In investigating the publication bias of studies included in the meta-analysis, a funnel diagram and Egger’s regression-based test were employed. The appearing funnel plot in Figure 3 did not indicate any clear publication bias in this meta-analysis, as studies were marshaled about the overall effect size in a symmetrical fashion. The geographic concentration and clustering of the findings toward the upper mean vertical axis suggest that the demographic studies that made it to the inclusion stage had more weight and fewer standard errors.

In order to check for publication bias, which is previously illustrated in Figure 3, an Egger’s regression-based test was also done, the results of which are shown in Table 4. Looking at the prevalence of publication bias in the meta-analysis, it can be observed that both the intercept and the standard error coefficient are not significantly different from zero. This confirms the results presented using the funnel plot in Figure 3.

**Table 4:** Egger’s regression-based test Egger’s regression-based test was used for the studies of Wilkie et al., 2022 [11], Chen et al., 2011 [12], and Lerman et al., 2013 [15]. SE: standard error; CI: confidence interval; t: test statistic from the linear regression analysis in which a significant t-value suggests symmetry evidence that can signify publication bias; p-value: the probability that the data observed has no publication bias in which if the p-value is low (less than 0.05), publication bias is more likely present.

Parameter	Coefficient	SE	t	p-value	95% CI	
					Lower	Upper
(Intercept)	0.015	0.1821	0.081	0.949	-2.300	2.329
SE	2.547	1.9291	1.320	.413	-21.964	27.058

**Figure 4:**
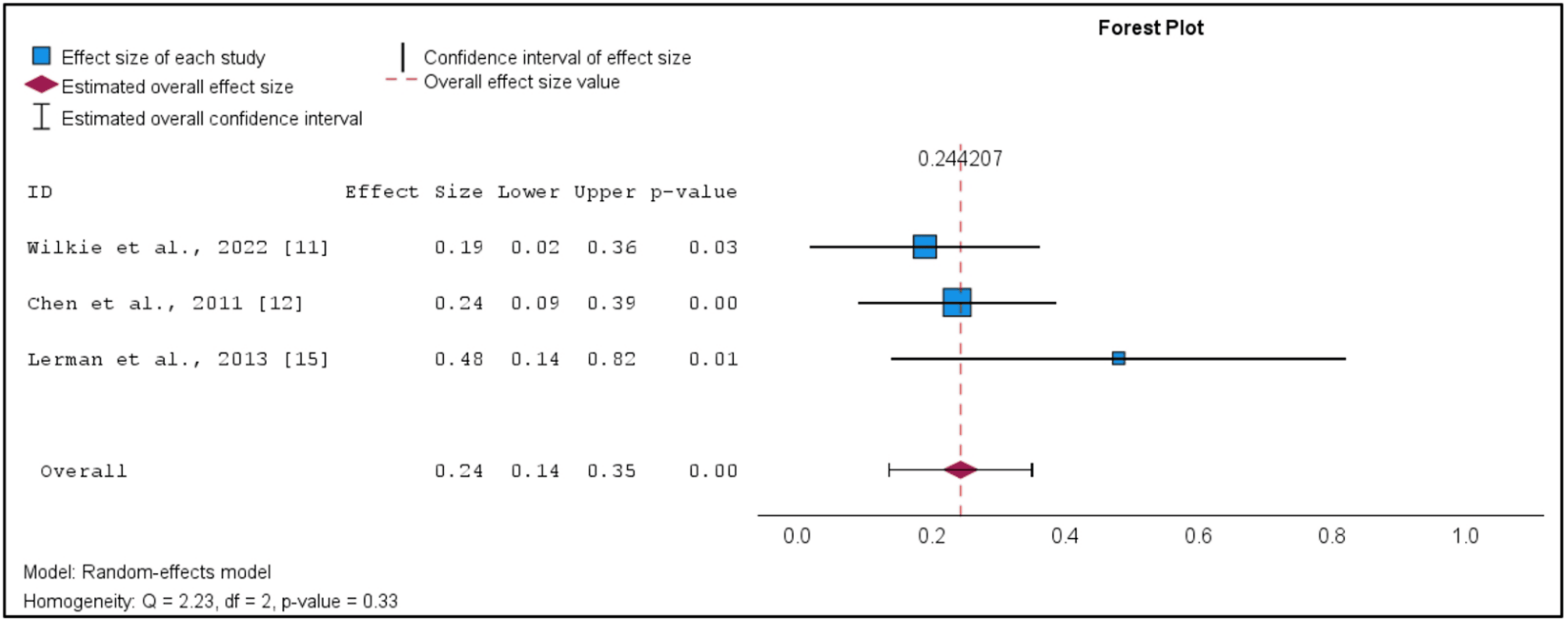
Forest plot on MVP and preterm delivery p-value: the probability that the data observed has no publication bias in which if the p-value is low (less than 0.05), publication bias is more likely present; z: critical value from the z distribution. MVP: mitral valve prolapse

The relationship between the MVP and premature delivery was assessed using random-effects meta-analysis with inverse variance weighting. The results are presented in Figure 4. All the ORs from the studies that were included were log-transformed for further analysis.

At the same time, the standard errors of the log-transformed ORs and their 95% confidence intervals were calculated using this formula shown here:

$SE = \frac{\log(\text{UpperCI})-\log(\text{Lower CI})}{2\times z}$

This formula has variables in which SE means standard error. Essentially, it tells us how precise our estimate is. Additionally, log(UpperCI)log⁡(UpperCI) means this is the natural logarithm of the upper limit of the confidence interval. Confidence intervals give us a range where we expect the true value of what we’re measuring to fall, most of the time. Furthermore, log(LowerCI)log⁡(LowerCI) means this is the natural logarithm of the lower limit of the confidence interval. Just like the upper limit, this helps define the range of possible true values for the measurement. Finally, zz means the z-score. This value tells us how confident we can be about our interval estimate. It essentially marks how many standard deviations a point is from the mean.

Figure 4 provides means to verify the presence of heterogeneity in the included studies (I² = 0%, p = 0.33) and such evidence is absent, which means that the effects’ size does not differ across the studies. The derived overall effect of the associations considered indicates significant z and p values of 4.47 and 0.00, respectively, meaning that there exists a significant relation between MVP and preterm delivery. The proof also thus reveals that preterm delivery is a disorder, whereas control patients suffer from MVP (pooled ES = 0.24, 95% CI: 0.14 to 0.35, p<.001).

Ethical considerations

In the course of this study, participant interaction was not needed, for this is a systematic review and meta-analysis that does not involve direct contact with patients. Entirely, we undertook the procedure of data systematic review by only surfing through scientific writings. We provided a modified syllabus on Good Clinical Practice (GCP) of great attention to detail upon implementing a study.

Discussion

Mitral Valve Prolapse (MVP) in Women of Reproductive Age

MVP is one of the common heart diseases when one or both of the mitral valve segments protrude into the left atrium during systole. The prevalence of MVP in the population has been reasonably well addressed, and estimates suggest it occurs in around 2-3% of people. Among the reproductive demographic, this ailment is particularly interesting as it may influence reproductive health. Where there are reports of a higher occurrence of the MVP among women, the relative male to female it is from 2:1 to 3:1. Such gender disparity in the incidence of this condition in women likely has to do with hormones, the structure of the heart, or the tendency to report to work [3,14]. In various population studies, MVP has been shown to have variations in frequencies in the various population groups.

According to the Framingham Heart Study, the proportion of women aged between 18 and 44 years of age who have this condition stood at 2.4 % [13]. Likewise, a similar study conducted among Turkish women of the same demographic found a proportion of 3.2% [16]. The findings are consistent with data from other states, including Europe and Asia, that disproves that MVP is not a problem among women at their reproductive age. Reasons for the differences in incidence could be due to differences in cultural, clinical, echocardiographic, and epidemiological factors. The course of MVP, usually benign in women of reproductive age, is often complicated in some cases. While a majority of patients are asymptomatic, other presentations of women with MVP might include palpitations, chest pain, and shortness of breath. Several studies have reported that a significant proportion of women with MVP also suffer from other conditions that put them at increased risk for poor cardiovascular outcomes, such as mitral valve regurgitation, endocarditis, and arrhythmias [1]. These complications may vary in their pattern of occurrence and severity, which could pose possible risks in pregnancy and need proper treatment and observation. Pregnancy outcomes in women with MVP have become the main focus of attention in recent times. Several reports have investigated the relationship between MVP and preterm labor due to more concerning aspects, which are the effects on the mother and the baby. Through a systematic and quantitative approach, Yuan et al. have found out that women with a diagnosis of MVP have an increased risk of preterm birth when compared to women without this condition [6]. It is crucial to prevent or treat women who are likely to conceive or are pregnant with MVP; if the latter is still stubborn to MVP, that is despite control of MVP.

It is necessary to continue investigations along such lines in order to explore the basic relationships that exist between MVP and undesirable pregnancy outcomes as well as to derive guidelines for the care of MVP patients who are pregnant. The existing recommendations stress the need for preconception consultation, scheduled cardiac monitoring, and individual approaches to therapy in order to improve both maternal and fetal health [23]. Further, and more, epidemiological work on this subject is likely to improve understanding of MVP, particularly with respect to the prevalence and impact on women of reproductive age.

Pathophysiology of MVP and its Impact on Pregnancy

MVP goes beyond the boundaries of a single ailment and encompasses many pathological processes. The highlighted point is that these individuals have a genetic predisposition to connective tissue diseases, which in turn create myxomatous changes in the mitral valve. Prior studies have noted the presence of extracellular matrix protein gene mutations, such as the fibrillin-1 gene, in the cases of structural defects found in MVP. Furthermore, the evolution of MVP has been attributed to environmental loading of the mitral valve, neurohormonal factors, and inflammation mechanisms [20]. These processes interrelate, causing a number of processes culminating in pathological remodeling of the mitral valve. Pregnancy is associated with various cardiovascular changes, and this may worsen some of the patient’s signs and symptoms in these patients with MVP. During pregnancy, as the blood and cardiac output increase, the mitral valve is under strain, which may enhance mitral regurgitation and elicit many symptoms such as palpitations, shortness of breath, and fatigue [24]. Also, there are alterations of hormonal levels during pregnancy that will particularly include high levels of estrogen and relaxin. This could contribute to the further myxomatous degeneration and prolapse of the valve leaflet [6]. These changes are new and require careful management of pregnant women with MVP to prevent complications. There is quite a plethora of other studies that have looked into the effect of MVP on pregnancy outcomes. Research indicates that women with MVP tend to have unfavorable pregnancy outcomes more frequently than women without this abnormality, such as preterm birth, fetal growth restriction, preeclampsia, etc. [19]. It was revealed in one large registrational study and meta-analysis that women with MVP are more prone to premature delivery than women without MVP [6]. The association between MVP and these adverse pregnancy outcomes is due to hemodynamic overload and the possibility of arrhythmias and heart failure. There is also the extension of this limitation whereby the availability of uterine artery perfusion may also limit the growth of the fetus. Abundant mitral regurgitation could also cause excessive blood toward the heart, as well as high lung capillary pressure due to excessive blood volume in the heart. This may further complicate the course of pregnancy and increase the risk to both maternal and fetal health.

To establish the best treatment protocol for MVP, especially during pregnancy, a multi-specialty team is required. Such a team will include, among others, cardiologists, obstetricians, and anesthetists. Counseling before conception is important for women with MVP because it helps assess the severity of the disease and plan for proper monitoring and management during pregnancy. Since pseudomacrostomy is not performed routinely, repeated echocardiographic studies are required in order to assess the degree of progression of MVP and the onset of mitral regurgitation. If an individual has severe mitral regurgitation of the mitral valve or congestive heart failure from symptomatic MVP, beta-blockers or other cardiovascular measures may be relevant [23]. This is a primary management within the primary disease prevention objectives. Care should be taken to highlight the fact that on some occasions it may be necessary to schedule surgery within a time frame prior to conception in order to repair or replace the mitral valve.

Association between MVP and Preterm Birth

Studies suggest that pregnant women with MVP face significant delivery challenges. Supported by that line of thinking, preterm delivery was attributed to factors such as blood supply disturbances combined with arrhythmias brought about by MVP [6]. The results of the inquiry into the management of pregnant women with MVP highlighted the importance of the prevention and control of preterm labor among this high-risk category of women. Another study was designed to examine the role of mitral regurgitation, which is often caused by the presence of MVP, in the anticipated obstetric results. It was found that the entity was associated with an increased rate of premature rupture of membranes in cases of moderate-to-severe mitral regurgitation. We suspected that the excessive regurgitation volume of the left atrium and ventricle inhomogeneous load may lead to functional decompensation of the heart and raise the risk of preterm labor [19]. Such findings underline the importance of performing detailed clinical and echocardiographic examinations to assess the degree of MVP and the degree of regurgitation in pregnant women. In Taiwan, population-based cohort studies provided further information approving the relationship between MVP theory and preterm delivery. The research focused on observing a subset of women who were already diagnosed with MVP and compared their pregnancy results to women without this diagnosis. It was reported that women who carried a diagnosis of MVP, on the other hand, had 1.27 times more chances of being preterm mothers. Such extensive research, in turn, proved that MVP is one of the prevailing risk factors, particularly in terms of the issues concerning preterm deliveries, and therefore management of such pregnancies should be enhanced [12]. However, apart from observational studies, Delling and Vasan, in a review done in 2014, demonstrated the mechanisms that rendered MVP in pregnancy to be associated with negative effects such as premiers. In the course of that study, however, it was brought forth that the hemodynamic stress as well as the neurohormonal activation experienced by those with MVP could lead to uteroplacental insufficiency, which is one of the main causes of premature birth [3]. Such findings necessitated the necessity of utilizing a multifaceted approach in caring for pregnant women with moderate MVP, especially those who had further complication risk factors such as diabetes or high blood pressure. In order to validate these results, a random-effects meta-analysis was carried out using the works of Wilkie et al., Chen et al., and Lerman et al. Collectively, these studies garnered data pertaining to 26,494 pregnant women with MVP [11,12,15]. Wilkie et al. employ a retrospective cohort study design that analyzes the Healthcare Cost and Utilization Project National Readmission Sample between the years 2010 and 2017. The study used codes from the ninth and tenth revisions of the International Classification of Disease to identify all pregnant women diagnosed with MVP. Research showed that MVP occurs in pregnant women and is associated with poor maternal cardiac status and obstetrical complications [11]. The analysis of Chen et al., in this regard, included birth and health outcome records utilizing the Taiwan Birth Registry and the National Health Insurance Research Dataset. In 2005, among pregnant women from Taiwan who delivered a single newborn, 3,104 moms were found to have MVP attending ambulatory or emergency care, while 12,245 mothers did not suffer any impact by the MVP. The research brought to the forefront that there was a considerable chance of early delivery in women who suffered from MVP. The investigation stressed the integration of a multidisciplinary team approach to the management of obstetric care in a bid to achieve appropriate care for maternal health. The principal objective of this method is to monitor the parameters of the cardiovascular system and preterm birth onset [12]. The research Lerman and co-authors conducted has been a retrospective comparison analysis scanning all singleton births during the years 1988-2010. The study analyzed women: those having mitral valve disorders (MVD) and those having no MVD. Women who did not receive any prenatal care were excluded. Several studies designed in such a manner pointed toward a link between MVD and the older age of the mother, repeated terminations of pregnancy, hypertensive diseases, and origin from a Jewish ethnic group. However, in this study, such information as the prevalence of patients with MVP was necessary, as they needed to be included in the analysis of other demonstrated factors [15].

Clinical Outcomes and Management of Pregnant Women with MVP

One of the difficult factors for pregnant women with MVP is the risk of worsening mitral regurgitation. This can create problems with excess fluid in the left atrium and the left ventricle. Increased pressure on the myocardium may result in breathlessness, fatigue, or palpitations. In some cases, it can even cause heart failure. The study, carried out by Van Hagen et al., had certain findings regarding the fact that the course of the most insidiously worsening complication of mitral regurgitation during pregnancy is a major factor predicting obstetric negative consequences such as preterm delivery and cardiac management after labor [18]. Therefore, routine echocardiographic assessment of the degree of mitral regurgitation must be performed for proper treatment strategy. Pregnancy increases the risk of sudden cardiac death, with emphasis on pregnancy-related heart disease. For women with MVP, arrhythmias may be a greater threat when they become pregnant. This may occur because a pregnant woman’s body undergoes physiological changes that may cause lower or higher blood pressure, such that pregnant women become at risk for irregular heart rates, for instance, atrial fibrillation and ventricular ectopy. Such embryos and newly delivered mothers in particular suffer the most serious cardiac events with their arrhythmic MVP [17]. Treating arrhythmias will mostly involve beta-blockers and antiarrhythmic drugs, which are harmful to the fetus and even the mother; hence, they should be selected properly. Management of MVP in pregnant women necessitates an organized approach involving obstetricians, cardiologists, and anesthesiologists. Additionally, women with MVP should attend preconception counseling to evaluate the degree of the risk and establish measures that will assist in the management of the pregnancy period. Hence, moving along in pregnancy, regular checkups are very important in determining the progression of MVP in pregnancy and tackling any cases that may arise. The use of beta blockers in MVP management is likely to be beneficial in decreasing complaints of palpitations and arrhythmias, and their use is consistent with pregnancy [21]. It may be indicated to perform surgery only for the cases of advanced mitral regurgitation that are symptomatic. However, doing this procedure during pregnancy is rare because of the associated risks that come along with it. Although this is less common, it is even more likely due to significant risks, particularly if the patient is pregnant. Unless severe signs and symptoms risk the mother’s life, then the operation is removed until after delivery because it is frequently delayed. In particular, a perioperative care delivery roadmap is relevant to patients who have either severe symptoms or severe mitral regurgitation during their pregnancy [25]. For such women with MVP, active management is required during the process of labor and for delivery as well. Taking the right kind of anesthesia and maintaining the hemodynamic state during the course of labor is critical in avoiding aggravation of mitral regurgitation or the occurrence of arrhythmias. As a rule, epidural anesthesia is the preferred method of anesthesia, as it facilitates effective pain relief and controls the blood pressure and heart rate sufficiently. Before the labor, it’s necessary to carefully debate but also come to an agreement with the obstetric and cardiac teams regarding the delivery plan. However, it is also important to have alternative strategies to deal with possible cardiac complications [26,22]. Additionally, an innovative modified Haller index (MHI) method was recently tested in healthy pregnant women. This index allows the clinicians to identify, among healthy pregnant women, those with a narrow anteroposterior thoracic diameter, causing ab-extrinsic cardiac compression, with increased prevalence of non-classic MVP, with mild impairment in myocardial strain parameters (particularly at basal level), in absence of any intrinsic myocardial dysfunction [27]. Further studies could be designed to evaluate if the MHI method might help clinicians to identify pregnant women with benign MVP and good prognosis.

Limitations

The small number of studies makes it difficult to apply the findings to a wider population, and the power of the results decreases. By virtue of the data set, the meta-analysis may not realize the range of characters and sources of variation that can be found in larger and more representative populations. The focus of the research was mainly based on studies, which is prone to selection and information biases as well as residual confounding. Although several confounding factors were assessed in the studies reviewed, there is still room for some of these factors to affect the results. Some of the studies may also bring in biases on the accuracy of subjects’ recollection of events and reporting of findings’ data. The non-inclusion of randomized controlled trials (RCTs) in this review suggests that it would be impossible to conclusively demonstrate the causal relationship. In this respect, whatever correlation is found, it must be emphasized that there is no absolute certainty regarding the nature of this correlation. Overall, clinical trials and prospective cohort studies are recommended to be more employed by such further studies to have a better picture.

## Conclusions

It has been shown that there is a sufficient association between the presence of MVP and the prematurity risk in females who are preparing for childbirth. In this case, the relevance of MVP in relation to consequences after childbirth should be stressed more so in focusing on the delivery process. In the case of patients with MVP, it is imperative that appropriate treatment strategies are sought due to the high incidence of birth prematurity in these patients. Such measures are ethically justified being in light of preventing further worsening of the MVP and the extent of valve incompetence. It is advocated that such a combination should include cardiologists, obstetricians, and anesthesiologists in order to optimize the outcomes for the mother and the baby. In other words, it is possible to say that it is necessary to establish a team-based approach to the care of women with pregnancy-related mitral valve regurgitation and arrhythmias including, but not limited to, beta-blockade. Further, optimal aims for perioperative management of women with pronounced valve regurgitation or symptoms will be aimed at enhancing safety in childbirth by developing individual-focused regimens. In the future, it will be very important to answer the questions of how and why MVP brings about adverse pregnancy outcomes and to formulate appropriate strategies for the management of these circumstances in the context of pregnant women.
